# Young patients with type 2 diabetes have high relative risks for complications in a country with middle-high sociodemographic index, similarly to those countries with high index

**DOI:** 10.3389/fendo.2025.1638565

**Published:** 2025-10-15

**Authors:** Gergő A. Molnár, Zoltán Kiss, István Wittmann

**Affiliations:** 2^nd^ Department of Medicine and Nephrology-Diabetes Centre, University of Pécs Medical School, Pécs, Hungary

**Keywords:** young people with type 2 diabetes mellitus, cardiovascular risk, myocardial infarction, stroke, mortality, renal risk, cancer risk

## Abstract

The absolute number of complications of type 2 diabetes mellitus increases with age. Recent data indicated that young individuals with type 2 diabetes are at a high risk compared to their non-diabetic counterparts, yet most data arose from high-income countries. Data in the literature indicates that young individuals indeed have a higher relative risk in terms of mortality as well as cardiovascular or renal events and cancer. We also present data on a set of nationwide analyses from Hungary, a middle-high-income country, a representative of the East-Central European Region, showing that the excess risk—when compared to non-diabetic individuals—is highest in the youngest age groups in terms of risk of mortality, myocardial infarction, stroke, dialysis, and also risk of cancer. We would like to raise the attention of caregivers to young individuals with type 2 diabetes who might be at a high risk as well.

## Introduction

Type 2 diabetes mellitus (T2DM) is a high-frequency disease in the general population with an increase in prevalence (1, 2). It leads to several chronic complications, including cardiovascular, cerebrovascular, lower extremity arterial disease, microvascular complications such as diabetic kidney disease (DKD), retinopathy or neuropathy, and also non-vascular diseases such as an increased risk of cancer or infections (3). Cardiovascular events tend to develop earlier in cases with T2DM as compared to individuals without T2DM (4). The absolute number of such events generally increases with age and is highest in individuals with T2DM and advanced age (5).

In a recent publication of Emerging Risk Factors Collaboration (ERFC) Study group, the life expectancies in young patients with type 2 diabetes mellitus (T2DM) were proven to be less favorable than in older patients (6). On the other hand, they stated that “The generalizability of the findings was enhanced by inclusion of data from 97 prospective studies from 19 high-income countries…” The Emerging Risk Factors Collaboration Study included data from the USA, the UK, Australia, Austria, Canada, Caribbean countries, China, Denmark, Finland, France, Germany, Iceland, Ireland, Israel, Italy, Japan, Netherlands, New Zealand, Norway, Scotland, Spain, Sweden, Turkey, and Venezuela; however, over 98% of individual data arose from high-income countries. The related editorial (7) also called for attention related to young individuals with T2DM but asked if the findings of the original study were similar in middle- or high-income countries. Moreover, there is only sparse data regarding this perspective of lower-income countries in general in the literature.

The aim of our review was to summarize the data of countries with higher sociodemographic index and also to provide data on Hungary as a country with high-middle sociodemographic index for comparison as a bit of answer to the question raised by the abovementioned editorial (7). Furthermore, we aimed to provide data not only on overall survival but also on most important components of mortality and morbidity, as they also severely impact the life of the individuals living with T2DM. In the present paper, we would like to draw attention to young individuals with T2DM and propose that these individuals are also at a marked risk and prove that they could have a higher relative risk compared to the non-diabetic population than the older patients with T2DM.

## Methods

### Search strategy and selection criteria

For the purpose of the narrative review, several PubMed searches have been run on articles published in English in the period 2000–2024 using combinations of the terms “diabetes mellitus” or “type 2 diabetes mellitus” or “diabetes mellitus (type 2)” and “risk” or “relative risk” or “hazard ratio” or “odds ratio” and “complications” and “cardiovascular” or “renal” or “kidney” or “nephropathy” or “AMI” or “stroke” or “cancer” and “age at diagnosis” or “young age” or “early-onset” or “young-onset”.

### Methods related to the studies from Hungary

The inclusion and exclusion criteria of the studies from Hungary as well as the detailed methods are provided in the original papers (8–13). Briefly, the data sources were the National Health Insurance Fund (NHIF) database and the Central Statistical Office (CSO) databases. The NHIF database covers mainly data related to diseases, medical diagnoses, interventions (such as coronary angiography, CT scans), and exact prescriptions of medications. The state insurance has nearly 100% coverage of the adult Hungarian population; thus, the NIF database enables a nationwide analysis. Therefore, data of all cases with T2DM and the entire population without DM can be compared. Furthermore, it also enables the use of matched control groups. Codes of the International Classification of Diseases (ICD) and data related to prescriptions of antidiabetic agents were used to set the diagnosis of T2DM as detailed in our original papers. Briefly, from the total population with diabetes, cases with gestational diabetes and polycystic ovary syndrome as well as cases with type 1 diabetes mellitus were excluded (12, 14–20). In our analysis of the period 2010–2013, data of more than 150,000 cases and more than 300,000 age-, gender-, and zip-code-matched controls could be compared in a 1:2 ratio manner (8, 9). The risks of mortality, acute myocardial infarction (AMI), stroke, and dialysis were analyzed, respectively. Because of the high costs, each individual dialysis case and event is captured by the NHIF database. Hemodialysis and peritoneal dialyses events were likewise taken into consideration. Event-free survival was tested in Cox regression models. As for the risk of cancer, in the studies, a population with incident T2DM of approximately 50,000 cases and a population with prevalent T2DM of approximately 600,000 cases could be compared to a non-diabetic adult population of approximately 7,000,000 cases (10, 11). In the studies on cancer, data were analyzed in the years 2015–2018 time frame, while we excluded cases diagnose with cancer in the preceding 5 years (i.e., 2009–2014) in order to try to exclude the recidivism of a previous cancer. Also here, a logistic regression was run for the analysis (10, 11).

## Results

### Epidemiological data related to age distribution of T2DM

Based on data of the IDF Atlas (2021 Edition), the worldwide number of cases with diabetes in the 20-24-year-old group is approx. 10 million, that of the 25-29-year-old-group is approx. 17 million, that of the 30-35-year-old group is approx. 25 million, that of the 35-39-year-old group is approx. 35 million. Summing up, the <40-year-old group could account for over 80 million cases worldwide. The 40-49-year-old age group could account for further 100 million cases worldwide (1). In the UK, the incidence of T2DM diagnosed at the age < 40 years, rose from 239 to 1541/100,000 individuals (21). The 2019 Global Burden of Disease (GBD) Study found that the incidence of individuals with T2DM in the 15–39 years age-range increased from 117.22 (95% CI: 117.07-117.36)/100,000 population in 1990 to 183.36 (95% CI: 183.21-183.51)/100,000 population in 2019 (2). Other data also indicate a rise in incidence of youth-onset T2DM that also accounts for an increasing burden for the society (22).

We have to mention, that the definition on young-onset T2DM or youth-onset T2DM or early-onset T2DM is quite heterogenous. Most studies refer to a <40-year-old adult population (6, 23–29), others use other cut-offs, some also include adolescent populations, as well (2, 30). Also, some papers compare <40 vs. >40-year-old groups (25), others a <40 vs. >60-year-old group, some show more detailed data in groups of decades of age (6, 8–11), thus the literature is quite heterogenous in that regard, as well. In the present review, we tried to capture data on young adults with T2DM, in the age of 18 to 39 years.

### The role of long duration and young age at diagnosis of diabetes as a risk factor of developing complications

When talking about the development of complications in young individuals with T2DM, it is hard to disclose the effect of diabetes duration on the risk of complications. With a long observation period, the younger the patient developed T2DM, the longer the duration will be by the end of the observational period. Unfortunately, in the literature, terms of young individuals and young-onset individuals are frequently used interchangeably, here, lower age at observation shall in fact be accompanied by a lower diabetes duration. Actually, the use of terms is more problematic in case of the older individuals with T2DM, as here, older individuals have frequently also a longer duration of diabetes, but since T2DM frequently develops also in more advanced age, some older individuals may have a short duration of diabetes. Diabetes duration is a known risk factor of developing complications (5), thus, in the present review we intended to find data related to age itself.

### Data related to risk factors (including hypertension and dyslipidemia) and age

In a study from Hong-Kong, patients with younger age at diagnosis had higher HbA_1c_ slope, faster deterioration of glycemic control and reacted less to metformin-based glucose-lowering treatment (31). A UK primary-care database-derived dataset found that individuals diagnosed with T2DM at an age of 18–39 years had higher HbA_1c_, higher LDL-cholesterol and more frequent obesity and lower use of antihypertensives or statins (26). In a cross-sectional study, the percentage of patients with CV disease and hypertension without receiving an antihypertensive agent was significantly higher in <40 years-age at diagnosis vs. >40 years-age at diagnosis (26.3 vs. 16.8) (25).

Another UK-based analysis also found that younger (< 40 years) age at diagnosis of T2DM is associated with higher HbA_1c_, LDL-cholesterol, triglycerides and obesity as compared to individuals with higher age at diagnosis of T2DM (28). A US-based analysis found a decrease in global longitudinal strain in young individuals with T2DM or with obesity as compared to controls, and this showed an association with age. The authors proposed that young-onset T2DM or obesity could predispose to the development of heart failure (32). According to a review, in general, a change in beta cell mass and obesity may contribute to early onset of T2DM (21). Besides, in a meta-analysis age-stratified analysis showed slightly higher adherence to oral antidiabetic drugs in older T2DM patients, with rates ranging from 49% in the youngest group to 58% in the oldest, although this trend was not statistically significant (p = 0.14) (33). In one of our earlier papers (14), we also found age-related differences in statin medication adherence in Hungary, where the younger cohort presented significantly lower, 7.9%-11.8% adherence during 12 months, while rates of adherence were 15.9% to 23.5% in older cohorts. Overall, age seemed to influence adherence more than gender, though the results did not reach significance, consequently, worse adherence to OADs may also play a relevant role in higher risk of diabetes complications.

### Data related to risk of mortality and age

The earlier mentioned study analyzing data of the Emerging Risk Factors Collaboration project found a higher hazard ratio (HR) values of mortality in patients diagnosed with T2DM at young age [30–39 years, 2.69 (95% CI 2.43–2.97)], while it decreased with age 40–49 years, 2.26 (2.08–2.45); 50–59 years, 1.84 (1.72–1.97); 60–69 years, 1.57 (1.47–1.67) and 70+ years, 1.39 (1.29–1.51) (6). The majority of cases of death were non-cardiovascular and were not related cancer, either (7). Despite worse status of cardiovascular risk factors in the young, the UK primary care-based analysis found similar mortality in different age groups (26).

### Data related to risk of macrovascular complications, cardiovascular disease, and age

In a study from China, the OR of developing CVD was 1.72 (1.36-2.17) in patients diagnosed with T2DM at the age of <40 as compared to those diagnosed with >60 years of age. The earlier T2DM was diagnosed, the higher was the odds of developing CVD (14% OR increase for every 5 years decrease in age) (27).

Worse glycemic control was associated with micro- and macrovascular complications [HR, 1.389 (1.163-1.658) and 1.252 (1.110-1.413), respectively] in individuals with young-onset diabetes, but not in middle-age-onset diabetes or late-onset-diabetes in a study from Korea (29). One study found a higher CVD risk in younger-onset T2DM vs. older-onset T2DM [hazard ratio: 1.48 (1.17-1.88)] (34). Research data from Singapore suggest that early-onset (<40 years) T2DM is associated with an 1.91 (1.19-2.40) hazard ratio of incident heart failure as compared to usual-onset T2DM. Adjustment for traditional risk factors had only a mild impact on the association, while adjustment for renal parameters abolished the association (35).

One study investigated data from Australia related to overall and cardiovascular mortality at different age at diagnosis of T2DM and found that there is an excess risk of CV mortality associated with earlier age at diagnosis, but this was mainly the effect of diabetes duration (36).

### Data related to risk of diabetic neuropathy and age

Another study, using propensity score matching, has found higher odds ratio in early-onset vs. in late-onset T2DM (<40 years vs. > 40 years) diabetic polyneuropathy (OR: 1.672), however, the risk disappeared when correcting for duration of diabetes (34). In the study, duration of T2DM especially that of >20 years had a marked effect, but in a logistic regression, also age of T2DM onset was associated with CV complications, the risk of neuropathy was only associated with age in the <40 year-old at diagnosis group, not the >40 year-old group (25). In a follow-up study of patients with a long duration (11.6+/-9.6 years for young-onset T2DM and 7.2+/-6.8 years for later-onset T2DM) form Korea, the risk of neuropathy was higher in the younger age group [OR: 3.05 (2.57-3.61)], and this has remained significant, even after correcting for duration of diabetes [OR: 1.39 (1.13-1.71)] (37).

### Data related to risk of diabetic retinopathy and age

Young-onset T2DM (<40 years) is associated with a high risk of diabetic retinopathy, especially in association with diabetic kidney disease, even with a duration of diabetes of only 3.7 ± 4.3 years (38). Another study also found a higher risk of retinopathy (2.906) in younger cases with T2DM (39). In the already mentioned study from Korea of patients with a longer duration (overall 8.0 ± 7.5 years) of diabetes, the risk of retinopathy was higher in the younger cohort [OR: 2.68 (2.14–3.35)], but this was mainly the consequence of a longer duration of DM, as after statistical correction for the duration the excess risk disappeared [OR: 0.98 (0.75–1.30)] (37).

### Data related to risk of diabetic kidney disease and age

Addressing kidney disease in epidemiological studies may be a hard issue, as various definitions and various endpoints could be used. Diabetic nephropathy is nowadays rather regarded as a histopathological diagnosis, and especially in individuals with T2DM, diabetic kidney disease (DKD) or chronic kidney disease (CKD) in DM could be appropriate endpoints. However, the definition of CKD or DKD would rely on abnormal albuminuria/proteinuria and/or decreased GFR in larger-scale studies, which may be affected by substantial bias in testing and may not be available in nationwide analyses. Moreover, CKD may be severely underreported when relying on ICD codes (40). In some analyses, hard endpoints such as initiation of dialysis and/or renal-related mortality are rather investigated.

A study with a median follow-up of 7.1 years found that young-onset (<40 years) T2DM was associated with a 35% higher hazard of CKD when compared to late-onset T2DM, but—just like in the case of CVD risk—this has vanished after controlling for diabetes duration (34). In the Korean study, young-onset T2DM was associated with higher odds of CKD [OR: 2.98 (2.27–3.90)] as compared to late-onset T2DM even in a multivariate analysis. However, when further correcting for T2DM duration, the excess risk disappeared [OR: 1.30 (0.93–1.80)]. Using cluster analysis, the same study found a higher percentage of severe insulin-deficient diabetes (46.4% vs. 22.7%) and less mild age-related (27.5% vs. 44.0%) and mild obesity-related (18.5% vs. 27.9%) diabetes within the persons with T2DM in the early-onset vs. the late-onset group (37).

A propensity-score-matched study found a higher risk of chronic kidney disease (CKD) (1.967) in younger-onset vs. older-onset T2DM (39). With regard to CKD, another study from Korea found that young-onset T2DM was associated with 1.70 (1.15–2.51) times higher odd as compared to late-onset T2DM. Within the young-onset group, hypertension, dyslipidemia, and sulphonylurea use determined the development of CKD (41). Data from Australia have shown that when investigating a combined endpoint of renal replacement therapy or renal-related death, young-onset T2DM was associated with a 2.0 (1.4–2.9) hazard ratio, even after adjustment for the duration of diabetes and other parameters. Adjustment for BMI weakened the association significantly (42).

### Data related to cancer

Since the prevention and treatment of acute complications and chronic, vascular complications have improved, we are faced with an increase of lifespan in our patients with T2DM. Thus, new long-term complications such as cancer or dementia are on the rise (3). Furthermore, data from the US suggest a rise in incidence of cancer in young individuals in general (these data are not related to T2DM specifically) between 2010 and 2019 [annual percent change, APC,+0.28% (+0.09–+0.47%)] (43).

A further paper presents data on newly diagnosed cases with T2DM with regards of cancer risk and age and found that the relative risk, expressed as standardized incidence ratio was highest [1.48 (1.41-1.54)] in the 20-54-year-old subgroup, decreased with the increase of age and turned even lower than 1.0 in the 76+ age group [0.86 (0.84-0.89)]. In that paper, data related site-specific cancer occurrence was also analyzed and similar result were found for individual cancer types (44).

Data from Australia show that cancer mortality (not incidence) rate ratio comparing young-onset vs. late-onset T2DM increased with increasing actual age; however, diabetes duration had a main influence on the data (36).

### Data arising for Hungary, a middle-high income country, related to the risk of mortality, cardiovascular and renal complications, and cancer in young individuals with T2DM

Hungary is found in the $50,000–100,000 income category along with, e.g., Poland, the Czech Republic, Slovakia, Croatia, and Romania (i.e., East-Central Europe) according to an analysis of Credit Suisse from 2021. For comparison, most countries participating in the ERFC Study are either in the $150,000–250,000 income category (such as Italy of Spain) or in the 250,000–350,000$ group (such as Austria, Canada, France, Germany, Iceland, Japan, New Zealand, the UK, Sweden, or Norway), in the $350,000–500,000 category (such as Australia, Belgium, Denmark, and Netherlands), or in the >$500,000 income category (such as the USA) (45, 46).

In addition, Hungary has ranked #52 in the list of countries according to wealth as opposed to the countries participating in the EFRC Study, where most countries rank higher than #25 (47). Thus, our data represents a country with a substantially lower income and wealth than that of the ERFC Study Group. Moreover, the GBD Study methodology also creates six so-called super-regions: the high-income super-region includes the US, Canada, Greenland, Australia, New Zealand, Brunei, Japan, Korea, Singapore, Argentina, Chile, Uruguay, and all countries from Western Europe. On the contrary, another super-region category was created to include Central Europe, Eastern Europe, and Central Asia, and this region includes Hungary (48). Thus, data related to Hungary may rather represent this super-region than that of high-income countries.

Between 2016 and 2024, our workgroup has published numerous analyses related to the field of epidemiology of diabetes mellitus in a program supported by the Hungarian Diabetes Association and based on data of the National Health Insurance Fund in Hungary (8–11, 13, 20, 49, 50). In general, the studies are based on data of NHIF and the CSO of Hungary.

According to our studies, upon the age pyramid of the populations, the relative frequency of the 18- to 39-year-old age group was 4.49% among patients with incident T2DM and 1.47% among patients with prevalent T2DM, while the frequency of the 40- to 49-year-old age group was 12.80% among patients with incident T2DM and 6.59% among patients with prevalent T2DM in Hungary (10, 11).

**Figure 1:**
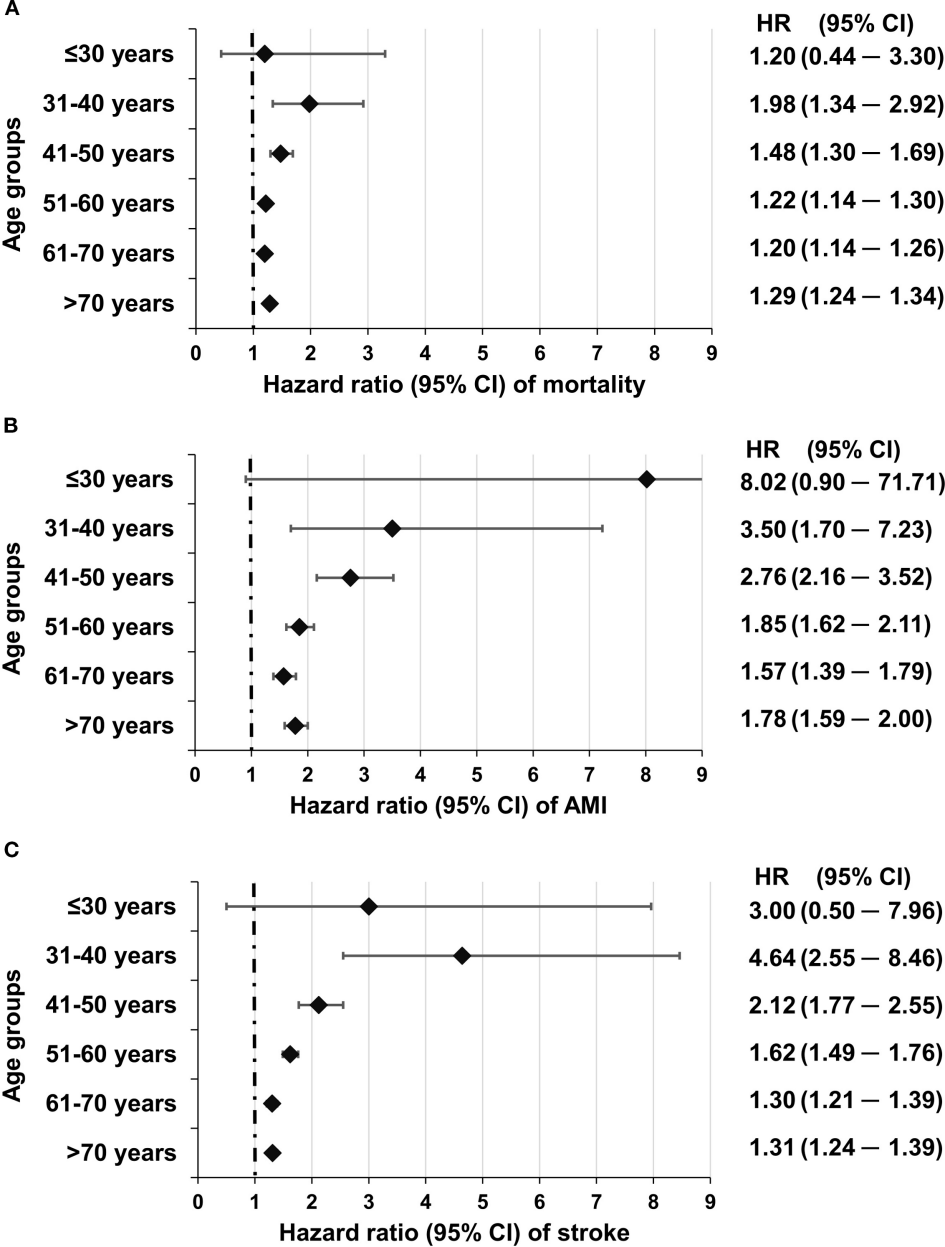
Risk of mortality **(A)** myocardial infarction **(B)** and stroke **(C)** in different age groups in individuals with T2DM as compared to individuals without diabetes from the same age group. Reproduced with permission from (8), under a Creative Commons 4.0 license.

Our workgroup has investigated mortality and cardiovascular risk in T2DM in a nationwide analysis in different age groups as compared to individuals without diabetes from the same age group. We found that the hazard of all-cause mortality was highest in the 31–40 group [HR, 1.98 (1.34–2.92)], and similar data were found for myocardial infarction [3.50 (1.70–7.23)] and stroke [4.64 (2.55–8.45)]. The hazards for all three outcomes decreased steeply with increasing age but remained significant even in the 70+ age group (**Figure 1**). Individuals with an age between 19 and 30 years had a low event rate. Thus, the HR values were too broad to achieve statistical significance (8).

**Figure 2:**
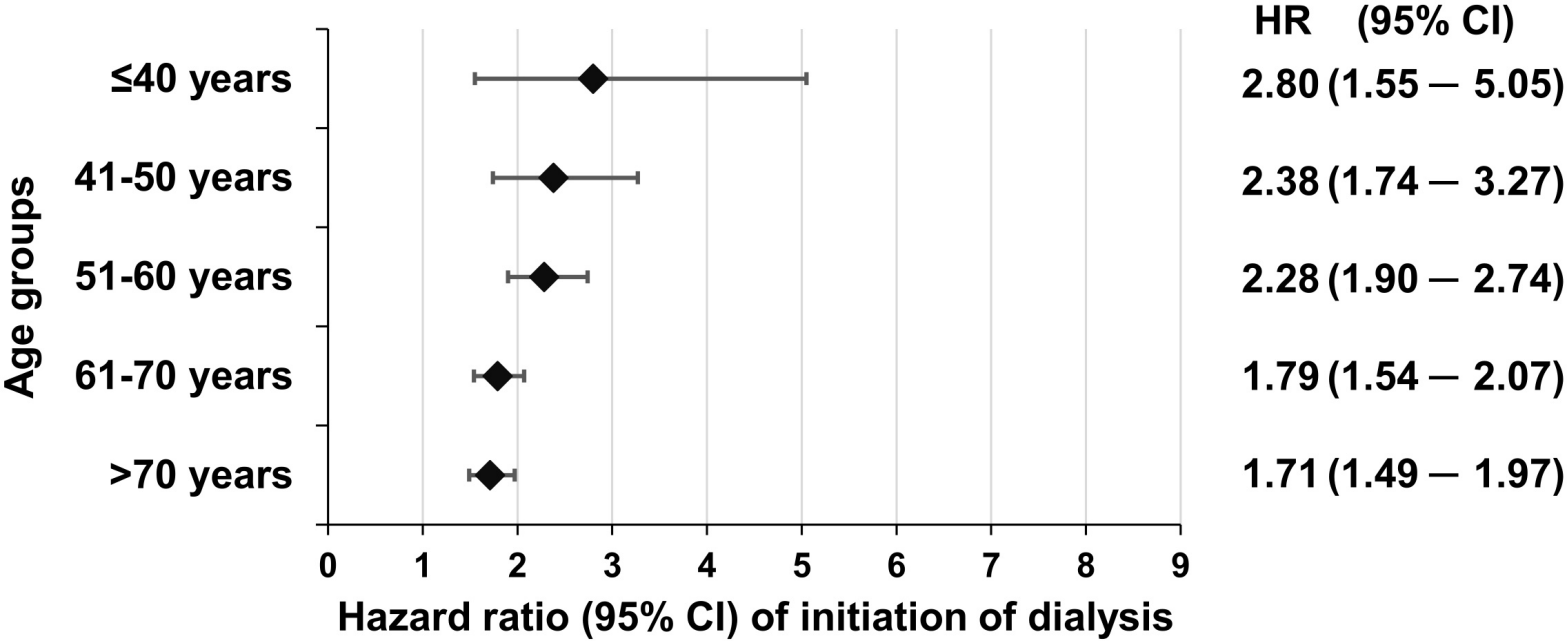
Adjusted hazard ratios related to the initiation of dialysis in individuals with T2DM in different age groups as compared to non-diabetic individuals. Reproduced from (9) with the permission of the publisher.

As for the risk of dialysis, the data of our workgroup have shown that there is also a significant (*p*<0.05) interaction between the hazard of dialysis and the age of the patients. Moreover, for this endpoint, the highest hazard ratio was found in the ≤40-year-old group [HR (95% CI), 2.80 (1.55–5.05)], which gradually decreased with increasing age [41–50 years, 2.38 (1.74–3.27); 51–60 years, 2.28 (1.90–2.74); 61–70 years, 1.79 (1.54–2.07)], and it was lowest yet still significant in patients >70 years of age [1.71 (1.49–1.97)] (9) (**Figure 2**). Furthermore, here we must acknowledge that the confidence interval of HR was widest in the youngest age group, yet the data of more than 11,000 patients were compared to the data of more than 23,000 subjects (T2DM vs. controls).

**Figure 3:**
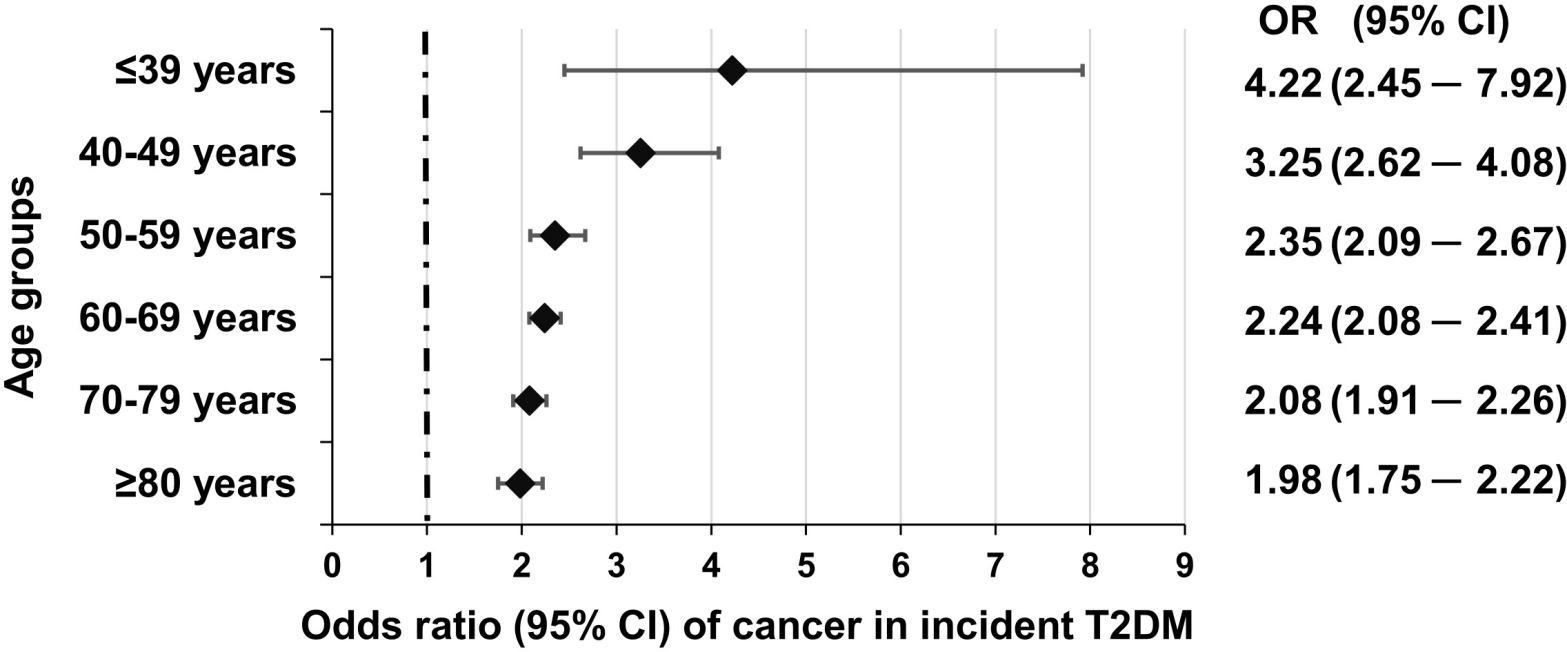
Odds of incident cancer in individual age groups in *incident* T2DM as compared to non-diabetic individuals. Reproduced from (10) under a Creative Commons license.

As for the risk of cancer, our workgroup found in a further nationwide study that in patients with *incident* T2DM (which is independent from duration of diabetes), when compared to more than 7,000,000 non-diabetic individuals, the risk of developing cancer was highest [OR, 4.22 (2.45–7.92)] in the 18–39 years old age group, and it was higher than in the 60–69, in the 70–79, or the 80+ age groups (**Figure 3**) (10).

**Figure 4:**
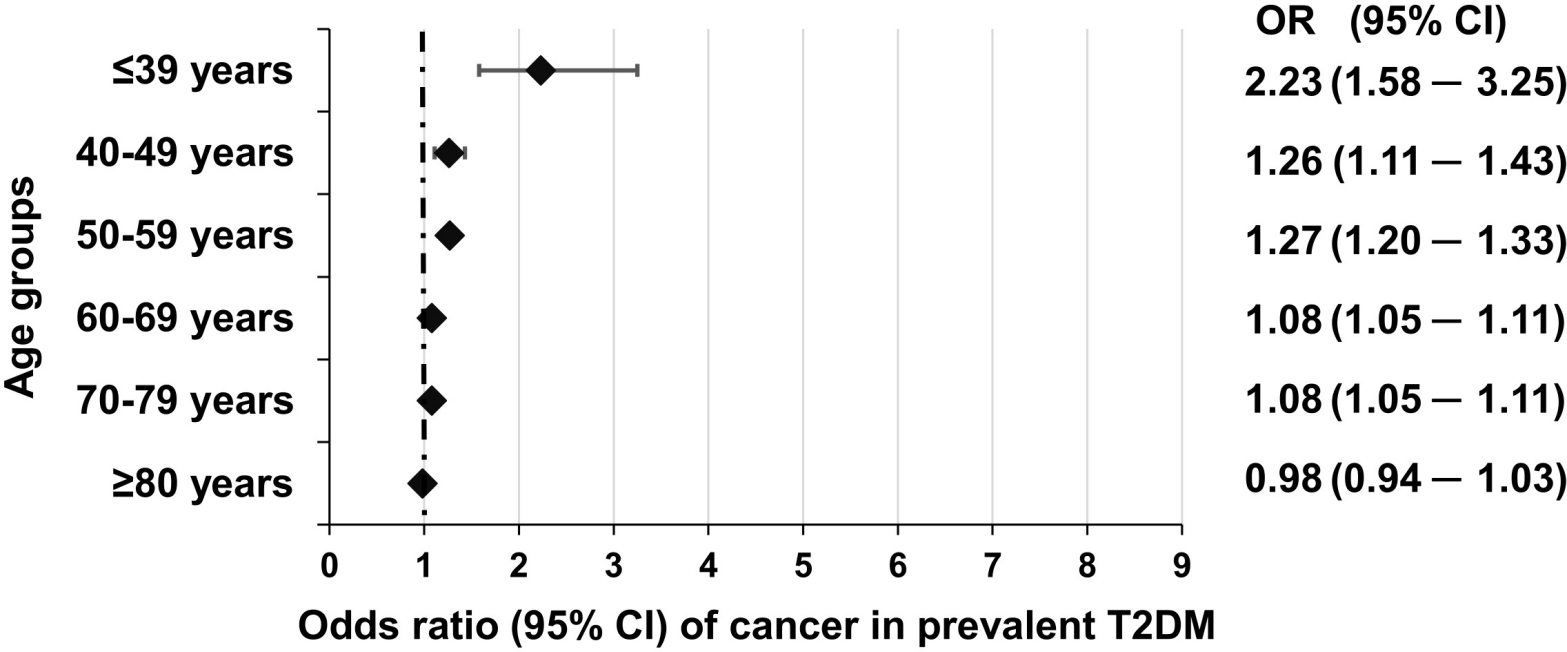
Odds of incident cancer in individual age groups in *prevalent* T2DM as compared to non-diabetic individuals. Reproduced from (11) under a Creative Commons license.

In another paper, we analyzed the *prevalent* cases of T2DM derived from the nationwide dataset, and also here we found a similar pattern than in the case of incident diabetes, the odds of developing cancer was highest in the 18–39 age group [2.23 (1.58–3.25)], and the odds ratio decreased with increasing age (11) (**Figure 4**).

### Pathogenesis of early T2DM

The pathogenesis of classical T2DM developing in middle-aged to elderly patients is well established; that of young-onset T2DM shares some characteristics but has also distinct features. The risk factors may include female gender, positive family history, obesity, gestational diabetes, maternal diabetes with *in utero* exposure, hypertension, dyslipidemia (51–55), and certain ethnic groups (such as Native people, Latino, Pacific Islander in the US (56) or Asian and Black people in England and Wales (57)). Obesity and prolonged insulin resistance may even be a stronger determinant of the development of early-onset T2DM than in the case of late-onset T2DM (22, 58–60). Puberty and related hormonal changes may affect the development of prediabetes in young adolescents and hence contribute to the development of early-onset T2DM (61, 62).

Data in the literature suggests that the fate of beta cells is different in early-onset vs. late-onset T2DM. Cases with early-onset T2DM may have a faster progression of deterioration of beta cell function (51, 52, 61); some data imply that the marked insulin resistance in these individuals further increases the loss of beta cell function (61).

In addition to genetic and metabolic factors, emerging evidence points to the role of psychosocial stress, adverse childhood experiences (63), sleep deprivation (64), and environmental endocrine-disrupting chemicals (65) in the early development of insulin resistance and T2DM in young people. These influences may contribute to unhealthy coping mechanisms (e.g., emotional eating), central adiposity, and chronic inflammation.

### Prevention of early-onset type 2 diabetes mellitus

The real primary prevention would be related to preventing the development of a manifest T2DM in high-risk young adults or even adolescents. A secondary prevention strategy could be used to reverse the development of T2DM and lead to remission in a patient who has already developed T2DM. A tertiary prevention aim could be the prevention of the development of complications of DM.

Non-pharmacological approaches may include patient education, diet (with omission of ultra-processed foods, high-carbohydrate-content meal, and soft-drinks), and exercise (62, 66). Of the pharmacological treatment possibilities, data related to metformin is rather controversial. Metformin has a label indication of use in case of prediabetes in adults, but not children or adolescents, and it is also indicated in polycystic ovary syndrome, another risk factor of the development of T2DM (62). However, the failure rate with metformin may be substantial (62, 67). Of the classical antidiabetic agents, a meta-analysis showed that pooled data of sodium-glucose transporter-2 (SGLT2) inhibitors empagliflozin and dapagliflozin indicate a protective effect against new-onset T2DM (62). In patients with prediabetes and obesity, GLP-1+/- GIP receptor agonists may be beneficial (68) also in young individuals (69), and recent data indicate that large doses of semaglutide led to a higher chance of normoglycemia (70), while large doses of tirzepatide led to a lower risk of new-onset T2DM as compared to placebo (62). Data also suggest that inhibitors of the renin–angiotensin–aldosterone system as well as supplementation with vitamin D might have a positive impact on the development of T2DM (68).

Beyond western medicine, traditional Chinese medicine (TCM) may also be an alternative way of preventing T2DM or its complications when used by a skilled person. Moreover, in TCM, non-pharmacological approaches such as specific types of exercise, emotional therapy, acupuncture, and nutritional approaches exist to try to prevent the development of T2DM (71). Furthermore, TCM gained attention in relation to specific medicines that could be used for delaying the progression by acting on glucose control, on inflammation, the microbiome, the glucagon-like-peptide-1 system, the glycogen metabolism, and the function of the beta cells. These medications may include Jinlida granule, Tianqi capsule, Tang-Min-Ling-Wan, Xiaoke Pill, or Jinqi Jiangtang. For a more profound overview, we suggest the review by Ni et al. (72) and Chen et al. (73), respectively. A meta-analysis suggests that Tianqi capsules may be effective in the prevention of T2DM (74).

### Young-onset T2DM and associated risk in male vs. female subjects

A detailed discussion of this topic is beyond the scope of the review, but we must admit that data is controversial. Some data suggest that young-onset T2DM is more frequent in women than in men and relate this fact to the effect of puberty and/or polycystic ovarian syndrome (21, 61, 75, 76). However, data from the US suggest that during adolescence, men may be more frequently affected by T2DM than women (22). The prevalence increases in both men and women worldwide, with a numerically higher increase in men, and in 2021, the number of cases was slightly higher in men than in women according to the Global Burden of Disease (GBD) Study (77). Furthermore, the age-standardized incidence was approximately 30% higher in the GBD study (2).

As to the complications, according to our studies, the hazard ratio related to the mortality of women with T2DM [1.37 (1.31–1.42)] was higher than that of men with T2DM [1.17 (1.12–1.21), *p*<0.001] when compared to women and men without T2DM. The HR of AMI was not different for women vs. men with T2DM [1.77 (1.59–1.98) vs. 1.83 (1.68–2.00), *p*=0.64), but the risk of stroke was again higher in women than in men with T2DM [HR: 1.14 (1.40–1.55) vs. 1.33 (1.26–1.41), *p*=0.01] (8). Concerning the risk of dialysis, the HR was higher in men than in women [2.21 (1.94–2.52) vs. 1.91 (1.68–2.17), *p*=0.031] (9). In cases with incident T2DM, the risk of cancer was similar in women and men with a tendency of favoring men (10). As for prevalent T2DM cases and cancer, the risk was significantly higher in men than in women [OR: 2.76 (2.76–2.82) vs. 2.27 (2.22–2.33), *p*<0.05] (11).

It is important to note that women generally have a lower baseline cardiovascular and mortality risk in the general population compared to men (78). Therefore, since the absolute risks of complications or death are similar or even moderately elevated in women with T2DM, the relative risks (e.g., hazard ratios vs. non-diabetic controls) may appear higher than in men. We could say that T2DM annihilates the relative protection seen in healthy women. This well-known epidemiological phenomenon may partly explain the seemingly higher excess risk observed in female patients with T2DM, especially in outcomes like all-cause mortality and stroke (8).

### Young-onset T2DM worldwide

The regional distribution of cases with young-onset T2DM is heterogenous. According to the Global Burden of Disease 2019 study, while the incidence of young-onset T2DM was highest in countries with low-middle to middle incomes, the related mortality of young-onset T2DM was highest in low-income countries. This may be related also to a high variance in the geographical distribution of risk factors such as the high consumption of processed or red meat, low consumption of fruits and whole grains, and differences in smoking habits (2). According to data from the IDF, the prevalence of young-onset (<40 years) T2DM was highest in the Western Pacific region, followed by Southeast Asia, the Middle East and North Africa region, and Africa, and it was substantially lower in Europe, North America and the Caribbean region, and South and Central America region (21). Moreover, the increase in prevalence was most notable in the Western Pacific Region, followed by Southeast Asia and the Middle East and North Africa region. There was a much lower rate of increase in North and South-Central America, while the prevalence was more or less stagnant in Europe (21).

## Conclusions

As for the general population, data indicate that while trends related to cardiovascular risk in elderly or middle-aged individuals are beneficial, on the contrary, young individuals have constant or even slightly increasing trends in the rate of events (79). A narrative review summarized evidence related to age and risk factors in the general population and strengthens the previous observation (80).

The data summarized in the present review indicates that young individuals do have a high risk of developing complications. In epidemiological studies, it is hard to distinguish the role of age, age at diagnosis, or diabetes duration as risk factors. The longer the follow-up, the more the results may be influenced by diabetes duration. Therefore, studying cases with incident T2DM is of excess value.

We have to emphasize that the absolute risk and the number of cases with complications is increasing as the age increases, but the relative risks or hazards may be highest in individuals with lower ages, as the number of events is relatively high in individuals with T2DM in these age groups. At the same the time, the number of events is low in the background population in the same age groups.

We presented a complex dataset from a middle-high income country supporting the excess risk of young individuals with T2DM in terms of overall mortality, the risk of myocardial infarction, the risk of stroke, the risk of dialysis and, as a novelty, also the risk of cancer. Our observations provide unique data for an East-Central European country.

Summing up, we believe that we should broaden our perspective related to T2DM and focus on young individuals as well. We should try to implement strategies not only to prevent the development of T2DM in young individuals but also to screen for complications in young cases with T2DM.

### Limitations

The present study was aimed at describing the risks associated with young-onset T2DM. It is not a systematic review, and no formal meta-analysis was carried out. Related to the data from Hungary, a clear limitation is that data of only one country were analyzed. However, we do not have access to data from any of the neighboring countries. We are not aware of another set of epidemiological studies from other countries in Central Europe as the set of more than 20 papers from the analyses of Hungarian data introduced above. The studies have been published between 2018 and 2024, cover the time frame 2009–2020, and represent not only one study but a set of studies arising from the same database system, on different time periods, with different populations, and of different methodologies.

A further limitation is that the NIF database does not cover any anthropometric parameters, laboratory data, nor data related to smoking, a clear risk factor in terms of cardiovascular, renal, or malignant diseases. Thus, comparisons could not be corrected to these factors.

In addition, the effect of the duration of diabetes cannot be ruled out in the case of studies on cases with prevalent T2DM. However, the analysis on cancer risk in individuals with incident T2DM rules out the contribution of longer duration to the excess risk in younger patients with T2DM.
